# Numerical Estimation of SAR and Temperature Distributions inside Differently Shaped Female Breast Tumors during Radio-Frequency Ablation

**DOI:** 10.3390/ma16010223

**Published:** 2022-12-26

**Authors:** Arkadiusz Miaskowski, Piotr Gas

**Affiliations:** 1 Department of Applied Mathematics and Computer Sciences, Faculty of Production Engineering, University of Life Sciences in Lublin, Akademicka 13 Street, 20-950 Lublin, Poland; 2 Department of Electrical and Power Engineering, Faculty of Electrical Engineering, Automatics, Computer Science and Biomedical Engineering, AGH University of Science and Technology, Mickiewicza 30 Avenue, 30-059 Krakow, Poland

**Keywords:** computational modeling, RF ablation, anatomical models, breast cancer, tumor shapes

## Abstract

Radio-frequency (RF) ablation is a reliable technique for the treatment of deep-seated malignant tumors, including breast carcinoma, using high ablative temperatures. The paper aims at a comparative analysis of the specific absorption rate and temperature distribution during RF ablation with regard to different female breast tumors. In the study, four tumor models equivalent to an irregular tumor were considered, i.e., an equivalent sphere and ellipsoid with the same surfaces and volumes as the irregular tumor and an equivalent sphere and ellipsoid inscribed in the irregular tumor. An RF applicator with a specific voltage, operating at 100 kHz inserted into the anatomically correct female breast, was applied as a source of electromagnetically induced heat. A conjugated Laplace equation with the modified Pennes equation was used to obtain the appropriate temperature gradient in the treated area. The levels of power dissipation in terms of the specific absorption rate (SAR) inside the naturalistically shaped tumor, together with the temperature profiles of the four simplified tumor models equivalent to the irregular one, were determined. It was suggested that the equivalent tumor models might successfully replace a real, irregularly shaped tumor, and the presented numeric methodology may play an important role in the complex therapeutic RF ablation process of irregularly shaped female breast tumors.

## 1. Introduction

Radio-frequency (RF) ablation is a reliable technique for the treatment of deep-seated malignant tumors using high ablative temperatures. A specific voltaged applicator operating at RF inserted into a tumor is usually the source of electromagnetically induced heat. The first step in planning the treatment usually involves positioning the body, skin marking, and taking imaging scans. The standard techniques used for breast cancer imaging include ultrasounds, mammography, magnetic resonance imaging (MRI), and positron emission tomography (PET), as well as techniques currently assessed at an experimental stage, such as microwave imaging (MI), infrared thermography (IRT), and others [1,2,3]. Early detection of neoplastic breast lesions and appropriate treatment at an early stage of the malignant disease significantly improve the chances of curing breast cancer, improve the quality of patients’ life, and facilitate a quick return to normal life [4].

Despite the use of advanced medical imaging techniques, the real spatial 3D shape and internal structure of breast tumors are not always known or properly visualized [5]. In the case of non-palpable cancers and various types of breast-conserving surgery (BCS) techniques, which involve surgical resection of the tumor itself with an appropriate margin of healthy tissue, it is usually assumed that female breast tumors have spherical shapes [6]. However, as shown by dissected breast tumors in patients after partial mastectomy as well as MRI scans [7], real cancerous tumors can exhibit a variety of sizes and complex shapes, including discoidal (flat and discoidal shapes, 34%), segmental (long and tubular shapes, 29%), spherical (19%), and other irregular shapes (16%) [5]. In this study, spherical tumors accounted for less than 20% of the analyzed cases. Wapnir et al. [8] observed an even smaller number of spherical tumors. They divided the shapes of female breast tumors into spherical (4%), oblate (18%), prolate (32%), and irregular (46%) tumors. In [9], the authors analyzed 22 MRI-based patient-derived breast repository models of tumors with different sizes and irregular shapes in the five basal areas of the female breast, i.e., upper outer, upper inner, lower outer, lower inner, and central breast locations. The tumor shape may vary depending on the patient’s supine (typical for surgery), erect, or prone position. Thus, most breast tumors have non-spherical shapes, and the knowledge of their exact shapes may allow surgeons to excise the breast cancer more precisely or apply another tumor-targeted therapy.

In many medical cases, it is not possible to resect the tumor due to anatomical limitations or tumor location, shape, and structure (multinodular tumors, dense vascularization, and infiltrative character) and other comorbidities; hence, other methods of cancer treatment are sought [10,11,12,13,14]. In recent years, minimally invasive ablative techniques for the treatment of inoperable tumors developing in various locations, including cancers of the liver, kidneys, lungs, bones, brain, and female breasts, have become very popular [15,16,17]. During RF and microwave ablation treatments, malignant cells are permanently damaged by increasing the temperature of the tumor tissue above the normal physiological threshold of the human body. The usual temperature ranges are 40–46 °C for hyperthermia (induction of apoptosis) and 50–100 °C for thermal ablation (permanent damage to proteins and induction of necrosis). An advantage of ablative techniques is their action at a specific point in the tumor upon the invasive insertion of a needle applicator into the target tissue, thus limiting the negative side effects in adjacent tissues [18,19,20]. The effectiveness of various thermo-therapies depends not only on the therapeutic temperature level but also on the design of the applicator, the frequency and the level of the input power, and the duration of the treatment [21]. Tumor temperature monitoring in real time during these medical procedures is still a challenge [22]. Most of the available hyperthermia and ablation systems use microwave frequencies of 915 MHz, 2.45 GHz [18], or higher [23]. Nevertheless, antennas with other frequencies [24,25,26], RF applicators [27,28], and RF coils [29] are designed as well. The number and arrangement of multiple puncturing applicators, often robot-assisted, is of great importance in the thermal treatment of the target tissue [13,30,31].

In computation practice, a spherical shape of the tumor is usually assumed in the case of hyperthermia and ablation procedures. These treatments are directed to the center of the tumor sphere, and the appropriate size of the treatment areas (ablation zones) is adjusted by changing the input power of the electromagnetic (EM) field applicators [32,33,34,35,36]. Similar simplifications are used in many other point-based cancer technologies, including those based on magnetic nanoparticles [37,38,39,40], where the tumor shape does not matter, but its location and size are important [41,42]. Various studies [43,44] investigated the influence of breast tumor shape on the microwave ablation treatment process. The use of antennas with a varied number of air gaps ensured better treatment of elongated tumors [20]. Additionally, the effect of micro-calcifications of female breast tissues on ablative treatment was considered in [45]. Non-invasive hyperthermia systems, where tumor temperature is regulated by an array of antennas surrounding the object, were tested as well [24,25,46,47,48].

During the numerical modeling of hyperthermia and thermal ablation, researchers most often model tumors using spheres [16,29,49,50,51,52,53] or ellipsoidal-like volumes [43,44]. However, the vast majority of invasive cancers are not spherical [5,9,54]; hence, the RF ablation of an irregular female breast tumor using a needle-type applicator with voltaged electrodes was analyzed in order to compare the modeling effectiveness. For this purpose, the temperature profiles, SAR distributions, and tissue power dissipation obtained in the naturalistic breast tumor model were compared with their spherical and ellipsoidal counterparts. To perform a detailed analysis of the problem, two scenarios of tumor counterparts were considered: (a) equivalent sphere S1 and equivalent ellipsoid E1, which have similar surfaces and volumes as irregular tumor T; (b) irregular tumor T inscribed into sphere S2 and ellipsoid E2 with correspondingly larger volumes.

## 2. Materials and Methods

In this section, the mathematical approach used in the electro-thermal coupling model, both in relation to the electromagnetic model (generalized Laplace equation) and the associated thermal model (modified Pennes equation), is described.

The commercially available Sim4Life software version 6.2 (Zurich MedTech AG, Zurich, Switzerland) as used for the simulations. It solves the described problem in Cartesian coordinates using two finite element method-based solvers. First, the Structured Electro-Quasi-Static Solver (EQS) was used to solve the Laplace equation, and then the specific absorption rate (SAR) coefficient was computed. Next, the SAR-based heat source was employed to estimate the temperature distribution in the breast model based on the modified Pennes equation. In this case, the Thermal Solver (TS) was applied. The calculations were performed using Intel (R) Xeon (R) CPU E5-26090, 2.40GHz with 64 GB RAM memory (Intal, Santa Clara, CA, USA).

### 2.1. Female Breast Phantom Model

**Figure 1:**
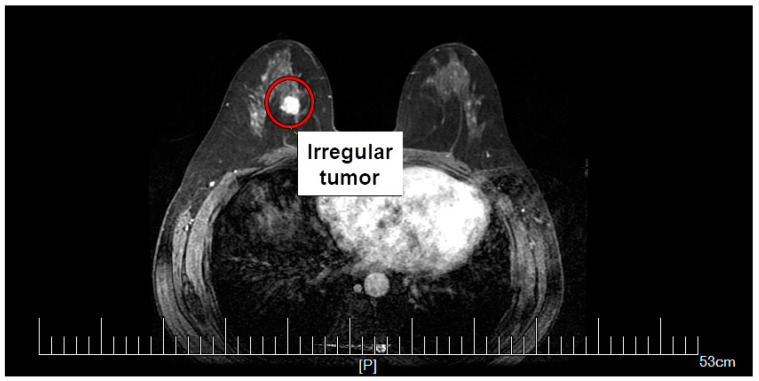
Screening mammography of the breast tumor. Reproduced with kind permission from Dalian University of Technology, China.

**Figure 2:**
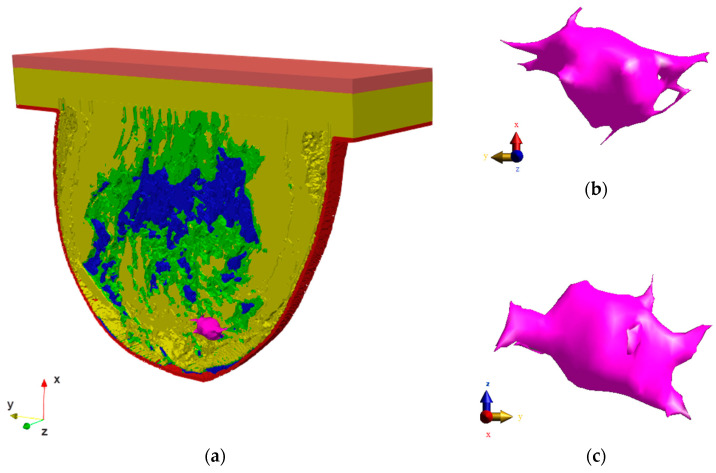
Views through (**a**) cross-section of the female breast phantom (*z* = 0) including the main tissues: breast fat (yellow), breast gland (blue), fat (green), muscle (orange), skin (red); and (**b**) irregular breast tumor (pink) oriented in the *x-y* plane and (**c**) oriented in the *y-z* plane.

To present the problem in the most realistic way, the calculations were performed using an anatomically correct model of the female breast with an irregularly shaped tumor. The breast tumor was screened at Dalian University of Technology, China. The mammography scan can be seen in Figure 1, together with the tumor marked with a red circle. From the mammogram, the irregular tumor was extracted and placed in the anatomically correct model of the female breast, as shown in Figure 2. The model of female breast tissues was adapted for numerical calculations from a breast phantom repository provided by the University of Wisconsin—Madison [55]. It consisted of skin, fibroconnective/glandular-1,-2,-3, transitional, fatty-1,-2,-3, as well as muscle tissues [9,29,56,57].

**Figure 3:**
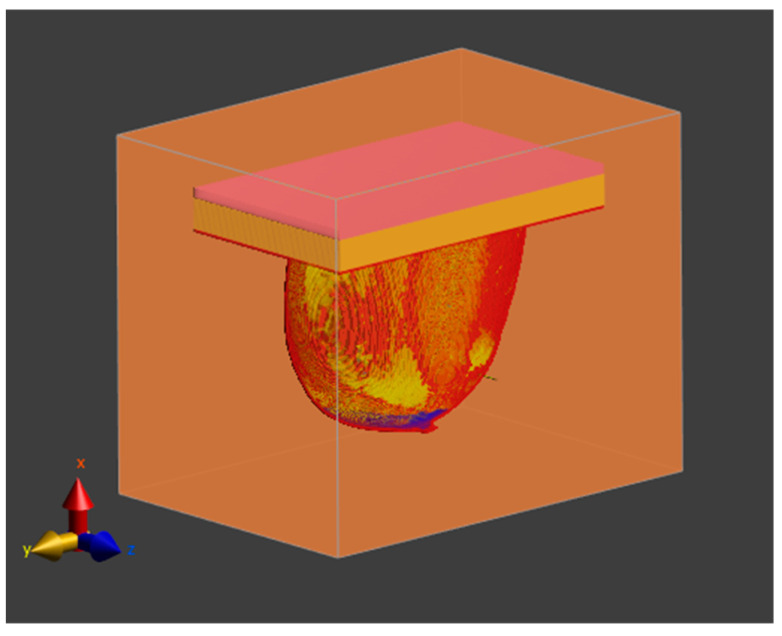
Computational area surrounding the female breast model.

The model represents a class 3 heterogeneously dense (HD) breast containing 51–75% of fibro-connective/glandular tissue [25]. It corresponds to the breast structure of a 35-year-old female patient. In this study, the breast fat parameters were set for the fatty-1,-2,-3 tissues, the breast gland parameters were set for the fibroconnective/glandular-1,-2,-3 tissues, the fat tissue parameter was set for the transitional tissue, and finally the muscle tissue parameters were set for the tumor. The whole model geometry of the analyzed female breast phantom including the irregular tumor oriented in different planes is shown in Figure 2, with the muscle tissue marked in orange, the glandular tissue in blue, the fatty tissue in yellow, the transitional tissue in green, the skin tissue in red, and the tumor in pink. The tumor was immersed in fatty breast tissue. The dimensions of the modeled tumor ranged from 10.87 mm to 42.59 mm and characterized small and medium tumors in the IA, IB, and IIA stages of breast carcinoma [58]. The whole model was surrounded by the boundary condition planes with dimensions of 411 mm × 528 mm × 380 mm that represent the background (air layer), as shown in Figure 3.

### 2.2. Material Properties

In this study, all modeled materials, including the female breast tissues and the RF applicator, are considered uniform, isotropic, and linear media, with no temperature dependence. Only the dielectric parameters of the tissues, i.e., electrical conductivity (*σ*), were considered to be frequency dependent and calculated for frequency *f* = 100 kHz, as reported in [59]. The nonlinear perfusion model, described by Equation (9), was considered only in the case of the tumor. Constant perfusion was assigned to the remaining tissues. Table 1 lists all the tissue parameters required for the in silico simulation, which were taken from the freely available Foundation for Research on Information Technologies in Society (IT’IS, Zurich, Switzeland) database of material properties [60]. Additionally, the dielectric components of the RF applicator (dielectrics and plastic catheter) were modeled using polyethylene material with an electrical conductivity of *σ* = 0.5 mS/m and a mass density of *ρ* = 1000 kg/m^3^. The electrodes were modeled as perfect electric conductor (PEC) materials.

### 2.3. RF Applicator Model

**Figure 4:**
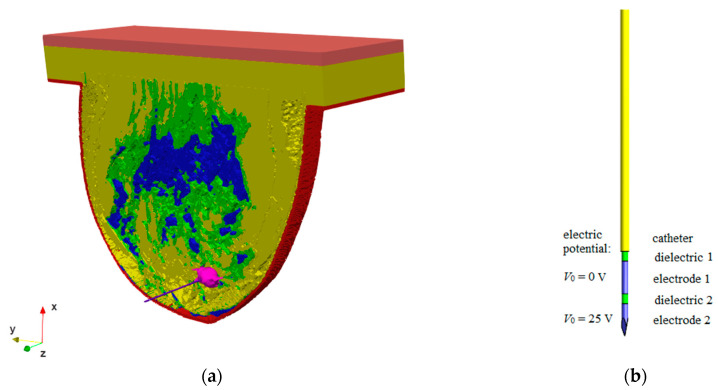
Model of (**a**) an RF applicator inserted into the irregular breast tumor, and (**b**) the internal structure of the needle including two electrodes in different electric potential conditions, dielectric, and external catheter.

Based on the available literature [27,49], an RF needle applicator with a diameter of 0.7 mm and a length of 70 mm containing two electrodes (5 mm in length and 0.5 mm in diameter) separated by dielectric 2 of the same size was used, as shown in Figure 4b.

The applicator was inserted in the *z*-direction into the female breast phantom with the irregularly shaped tumor, as shown in Figure 4a. The electric potential of *V*_0_ = 25 V was assumed on the lower electrode (electrode 2), whereas the upper electrode (electrode 1) was grounded (*V*_0_ = 0). The upper dielectric (dielectric 1) with a length of 58.5 mm and a diameter of 0.5 mm was surrounded by a plastic catheter measuring 57 mm in length and 0.7 mm in diameter, which served as a protective element.

### 2.4. Equivalent Tumor Models

**Figure 5:**
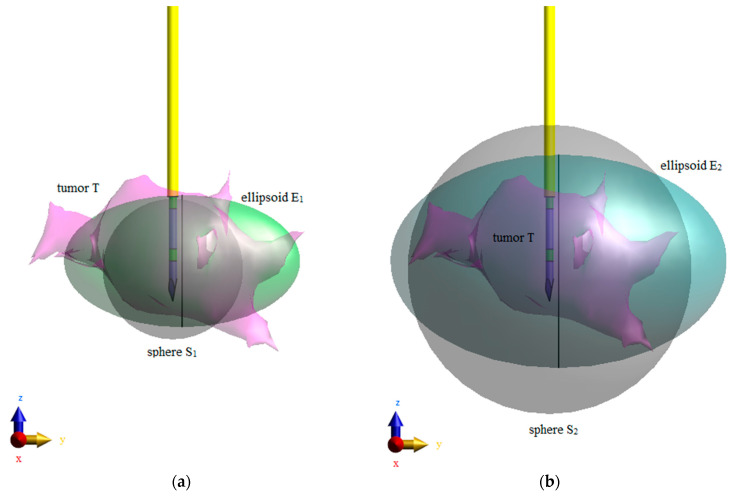
Analyzed scenarios of tumor shapes: (**a**) irregular tumor T, equivalent sphere S_1_, and equivalent ellipsoid E_1_ have the same surfaces and volumes; (**b**) irregular tumor T is inscribed into sphere S_2_ and ellipsoid E_2_ with correspondingly larger volumes.

In order to analyze the temperature profiles of tumors with different shapes, two cases of tumor shapes were considered, as shown in Figure 5. In the first scenario, irregularly shaped tumor T was compared to equivalent sphere S1 and equivalent ellipsoid E1, which had the same surfaces and volumes as the tumor (Figure 5a). In the second case, irregular tumor T was replaced with sphere S2 and ellipsoid E2, with correspondingly larger volumes surrounding the tumor (Figure 5b). The geometric parameters and masses of all analyzed tumor models are compiled in Table 2.

The data gathered in Table 2 present the largest tumor sizes *a*, *b*, *c* (along the *x*, *y*, *z* axes, respectively), which correspond to the diameter *d* = *a* = *b* = *c*, in the case of spheres or the longer axes in the case of ellipsoids. Besides, the parameters in the table allow comparison of the total surface areas (*A*), volumes (*V*), and masses (*m*) of individual models calculated using well-known formulas [43]. Some of the aforementioned parameters were measured and calculated based on the tumor model meshes (marked with an asterisk: *).

In the case of tumor T, the exact values of the geometrical parameters (*A*, *V*, *m*) were calculated for an equivalent cube with sizes *a*, *b*, *c* (values marked with a double asterisk: **) or measured from the mesh (values marked with a single asterisk: *). The analysis showed that the parameters calculated and measured for all analyzed shapes were consistent. However, ellipsoid E1 was the most similar to the actual tumor T in terms of the analyzed geometric parameters.

### 2.5. Electro-Conductive Field and Generalized Laplace Equation

The mathematical model, which describes the phenomenon of heat dissipation in human tissues together with the RF applicator, is based on a quasi-static assumption of electro-conductive field governed by the following formulas [26]:

(1) ∇⋅J=0

(2) J=σE

(3) E=−∇φ

where **J** and **E** correspond to the vectors of current density (A/m^2^) and electric field strength (V/m), respectively, *σ* stands for the electric conductivity of the material (S/m), and *φ* means the electric potential (V).

A quasi-static approximation can be assumed because the wavelength of the applied 100 kHz EM field (*λ* = c_0_/*f* ≈ 3 km) is much larger than the largest size of the analyzed RF applicator, and thus the displacement currents compared to the conduction currents are negligible [27]. Since the *E*-field pattern around the needle applicator is forced by the voltage applied to electrode 2 (see Figure 4b), the generalized Laplace equation in the following form can be used:

(4) ∇⋅(−σ∇φ)=0

To solve the described problem, the Dirichlet boundary conditions for electric potential (*φ*) were applied, i.e., electrode 1 of the RF applicator was grounded (*φ* = *V*_0_ = 0) and electrode 2 was voltaged by electric potential *φ* = *V*_0_ = 25 V (see Figure 4b); *φ* = *V*_0_ = 0 was assigned to the external planes of the computational domain. The other boundaries, which result from the EM field theory and reflect the continuity of the normal components of the current density vector between two adjacent tissues, can be introduced as:

(5) $$n\cdot \left(J_{1}-J_{2}\right)=0$$

or in the equivalent form as:

(6) $$n\cdot \left(\sigma_{1}\nabla \varphi_{1}-\sigma_{2}\nabla \varphi_{2}\right)=0$$

### 2.6. Modified Pennes Bioheat Transfer Equation

The modified Pennes equation with additional components is most often used in the modeling of heat flow in biological tissues. This model may predict temperature changes during hypothermia, hyperthermia, and ablation treatments, which are various kinds of thermo-therapy. It takes into account both the macroscopic interactions between the vascular system and the tissue, in particular the cooling effects on the blood flowing through tiny vessels, as well as the metabolic processes in living tissue [61]:

(7) $$\rho c\frac{\partial T}{\partial t}=\nabla \cdot \left(k\nabla T\right)-\mathrm{HTR}\left(T\right)\left(T-T_{b}\right)+\rho \mathrm{HGR}+\rho \mathrm{SAR}$$

where the first element corresponds to the heat accumulation inside tissues during the hyperthermia time *t* (s), *c* (J/kg/K) stands for the tissue-specific heat, and *ρ* (kg/m^3^) is the tissue mass density. The second term describes heat conduction in tissue with thermal conductivity *k* (W/m/K). The third term relates to the cooling effects of blood perfusion through the tissue expressed by the heat transfer rate HTR (mL/min/kg) as well as the difference between the current temperature of tissue *T* (K) and the arterial blood temperature *T*_b_ (K) [61]. The next term describes heat losses induced by tissue metabolism. This element is proportional to the heat generation rate HGR (W/kg). The last term, often called external heat generation *Q*_ext_ = *ρ*SAR (W/m^3^), describes heat losses caused by the RF applicator. The SAR-based heat source measures the EM energy absorbed by the tissue unit mass during unit time. The SAR (W/kg) parameter is proportional to the tissue temperature [25,62], namely:

(8) $$\mathrm{SAR}=\frac{d}{dt}\left(\frac{dW}{dm}\right)=\frac{d}{dt}\left(\frac{dW}{\rho dV}\right)=\frac{dP}{dm}=\frac{dP}{\rho dV}=\frac{\sigma }{2\rho }E\cdot E^{*}=\frac{\sigma }{2\rho }{\left|E\right|}^{2}=\frac{\sigma }{2\rho }{\left|\nabla \varphi \right|}^{2}\sim \frac{dT}{dt}$$

where *W* (J) means the electromagnetic energy with power *P* (W) deposited by the female breast tissue with volume *V* (m^3^), mass *m* (kg), and density *ρ* (kg/m^3^), |**E**| = |∇*φ*| (V/m) stands for the amplitude of electric field strength produced by the RF applicator voltaged by the electric potential *φ* (V), *σ* (S/m) is the electrical conductivity of the medium, and *t* (s) is the duration of the EM field exposure. The SAR parameter is a coupling of the modified Pennes bioheat equation (7) with the generalized Laplace equation (4) and plays an extremely important role in EM field dosimetry and human tissue safety [62,63].

The blood flow through breast carcinoma was defined by a nonlinear temperature-dependent blood perfusion rate to fully reproduce the dense vascularization of the breast tumor and its complex thermoregulatory processes [25]:

(9) $$\mathrm{HTR}\left(T\right)=\rho_{b}c_{b}\rho \omega \left(T\right)=0.4+0.4\exp\left(-\frac{{\left(T-37\right)}^{4}}{880}\right)\left[\frac{\mathrm{mL}}{\min\;\mathrm{kg}\;K}\right]$$

This element is proportional to the blood specific heat *c*_b_ (J/kg/K), blood density *ρ*_b_ (kg/m^3^), tissue density *ρ* (kg/m^3^), and blood perfusion *ω* (1/s); as the temperature increases, the tumor perfusion decreases exponentially [57].

The modified Pennes equation (7) should be completed by the proper boundary conditions. The heat flux on the skin tissue surface, coming from the external air environment, was modeled using the third kind (Robin) boundary condition [25,44]:

(10) $$n\cdot \left(k_{\mathrm{skin}}\nabla T\right)=h\left(T-T_{\mathrm{ext}}\right)$$

where *h* is the overall heat transfer coefficient modeling the coupled convective and radiative heat losses on the breast skin surface, *k*_skin_ (W/m/K) is the skin thermal conductivity of the breast phantom, *T*_ext_ stands for the air temperature that surrounds the breast model, and **n** relates to the normal vector perpendicular to the skin layer surface.

Since no contact resistance occurs between the internal breast tissues, the continuity of the heat flow within all the interior boundaries has to fulfill the relation:

(11) $$n\cdot \left(k_{1}\nabla T_{1}-k_{2}\nabla T_{2}\right)=0$$

where subscripts 1 and 2 in the equation correspond to two different sides of a given breast tissue internal boundary.

### 2.7. SAR and Power Dissipation Values

The local SAR defined by Equation (8) at a given location of the computational domain is not always useful during computer simulations, because it is too sensitive to approximation procedures in most computational methods. Moreover, the EM energy deposited at each tissue point (*x*, *y*, *z*) is often smeared out due to the occurrence of heat conduction; thus, the local values of the SAR coefficient are not thermally important [64]. There are many various definitions of SAR, which refer to total loss power *P* (W) and total loss power density *p* (W/m^3^) deposited in the target tissue region with complete mass *M* (kg) and volume *V* (m^3^). In general, two main approaches of SAR averages are commonly employed in numerical simulations, namely values averaged over some finite mass SAR_mass_ (W/kg) or volume SAR_vol_ (W/m^3^) as defined below:

(12) $${\mathrm{SAR}}_{\mathrm{mass}}=\frac{1}{m}\int_{M}\mathrm{SAR}\;dm=\frac{1}{\rho V}\int_{V}\rho \mathrm{SAR}\;dV=\frac{{\mathrm{SAR}}_{\mathrm{vol}}}{\rho }=\frac{p}{\rho }=\frac{P}{m}\;=\frac{1}{2m}\int_{V}\sigma {\left|E\right|}^{2}\;dV$$

(13) $${\mathrm{SAR}}_{\mathrm{vol}}=\frac{1}{V}\int_{V}\rho \mathrm{SAR}\;dV=\frac{\rho }{m}\int_{M}\mathrm{SAR}\;dm=\rho {\mathrm{SAR}}_{\mathrm{mass}}=p=\frac{P}{V}=\frac{1}{2V}\int_{V}\sigma {\left|E\right|}^{2}\;dV$$

where |**E**| = |∇*φ*| (V/m) stands for the amplitude of electric field strength produced by the RF applicator. These equations indicate that the averaged SAR values are scaled and related by the formula SAR_vol_ = *ρ*SAR_mass_. Knowing such values, it is possible to estimate power dissipation in the targeted tissue, including total power losses:

(14) $$P=\int_{M}\mathrm{SAR}\;dm=m{\mathrm{SAR}}_{\mathrm{mass}}=\int_{V}\rho \mathrm{SAR}\;dV=V{\mathrm{SAR}}_{\mathrm{vol}}=pV=\frac{1}{2}\int_{M}\frac{\sigma }{\rho }{\left|E\right|}^{2}\;dm=\frac{1}{2}\int_{V}\sigma {\left|E\right|}^{2}\;dV$$

and total loss power density:

(15) $$p=\frac{P}{V}=\frac{1}{V}\int_{M}\mathrm{SAR}\;dm=\frac{1}{V}\int_{M}\frac{\sigma }{2\rho }{\left|E\right|}^{2}\;dm=\frac{1}{2m}\int_{M}\sigma {\left|E\right|}^{2}\;dm=\frac{1}{2V}\int_{V}\sigma {\left|E\right|}^{2}\;dV=\rho {\mathrm{SAR}}_{\mathrm{mass}}$$

or in the equivalent form:

(16) $$p=\frac{P}{V}=\frac{1}{V}\int_{V}\rho \mathrm{SAR}\;dV=\frac{1}{2V}\int_{V}\sigma {\left|E\right|}^{2}\;dV=\frac{\rho }{2m}\int_{V}\sigma {\left|E\right|}^{2}\;dV=\frac{1}{2m}\int_{M}\sigma {\left|E\right|}^{2}\;dm={\mathrm{SAR}}_{\mathrm{vol}}$$

The presented derivations show that all the parameters described above are closely related and that the total loss power deposited inside a tissue is equal to the SAR_vol_ value. Additionally, the peak spatial-average SAR (psSAR) for constant-mass cubes of tissue (e.g., 1 g) is defined according to the IEEE/IEC 62704-1 standard [65].

## 3. Results

This section summarizes the in silico analysis results obtained by solving the conjugate electro-thermal problem. Moreover, the dosimetric analysis of local and averaged SAR parameters and peak spatial-average SAR (psSAR) are presented. Finally, time complexity is introduced.

The results of the application of the Structured Electro-Quasi-Static (EQS) Solver are summarized in Table 3, where the following data are compiled: the maximum local SAR values (SAR_max_), the SAR value averaged in unit mass (SAR_mass_) and volume (SAR_vol_) of various female breast tissues, including equivalent tumor models, as well as the SAR value averaged by 1 g mass of tissue (spSAR_1g_). Additionally, the total power losses induced inside individual female breast tissues are given according to the formulations in Section 2.7. The total mass and volume of the breast tissues and the values for the equivalent tumor models are included as well.

It can be seen that the maximum local SAR for the tumor models (E1, S2, E2, S2, and T) ranged from 152 to 167 kW/kg. The highest value of 167 kW/kg was reached for irregularly shaped tumor T, while the maximum local SAR was almost the same for the other models. The values of the mass-average SAR ranged from 59 to 328 W/kg, reaching the lowest value of 40 W/kg and 59 W/kg for S2 and E2, respectively, due to their much higher volumes. In this case, the values of the mass-average local SAR for T, S1, and E1 did not change considerably. An analogous result was exhibited for the total loss power density, which is simply the total volume-averaged SAR value. The values of both spatial-average SAR and total loss power did not change considerably and ranged from 823 to 825 W/kg and from 0.98 to 1.06 W, respectively.

**Figure 6:**
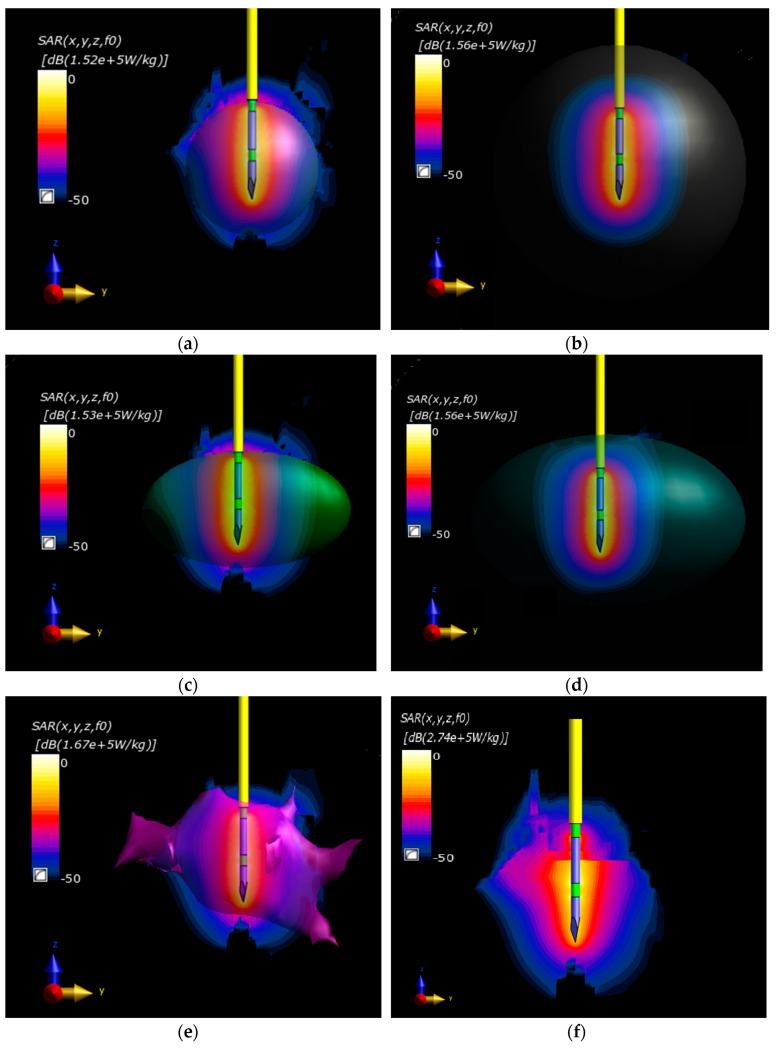
Normalized SAR distributions in the *yz* plane derived from the RF needle applicator for all analyzed equivalent tumor models: (**a**) sphere S_1_; (**b**) sphere S_2_; (**c**) ellipsoid E_1_; (**d**) ellipsoid E_2_; (**e**) irregular tumor; (**f**) case without tumor.

The SAR distributions in the considered scenarios are presented in Figure 6. The SAR distribution related to the maximum value for each tumor model was presented in the decibel scale for better visualization. The SAR distributions were combined with the rendered tumor model for comparison.

**Figure 7:**
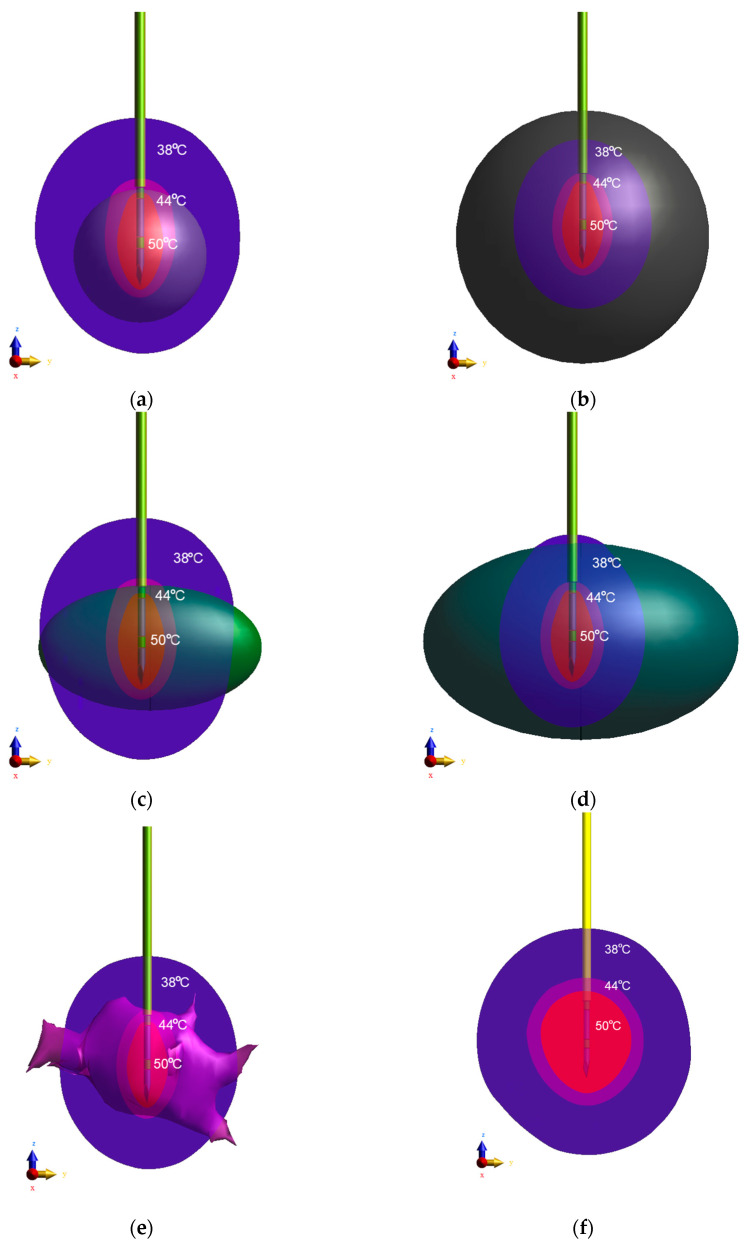
Isosurfaces of temperature in the *yz* plane derived from the RF needle applicator for all analyzed breast tumor equivalent models: (**a**) sphere S_1_; (**b**) sphere S_2_; (**c**) ellipsoid E_1_; (**d**) ellipsoid E_2_; (**e**) irregular tumor; (**f**) case without tumor.

Taking into account the SAR-based heat source and Pennes formulation, the temperature distributions were obtained by applying the Thermal Solver. The following coefficients were assumed: *h* = 5 W/m^2^/K, *T*_ext_ = 25 °C, and *T*_0_ = *T*_b_ = 37 °C [25,66,67], which correspond to the heat transfer coefficient, external temperature, and initial temperature, respectively. The induced steady-state isothermal surfaces for temperatures of 50 °C, 44 °C, and 38 °C (isosurface-50, isosurface-44, isosurface-38) obtained for the differently shaped tumors in the same RF applicator operating conditions (*f* = 100 kHz) are shown in Figure 7. Isosurace-50 is marked in red, isosurface-44 in pink, and isosurface-38 in blue. In this case, isosurface-50 was considered an ablation zone. Besides, the rendered equivalent tumor models were added.

**Figure 8:**
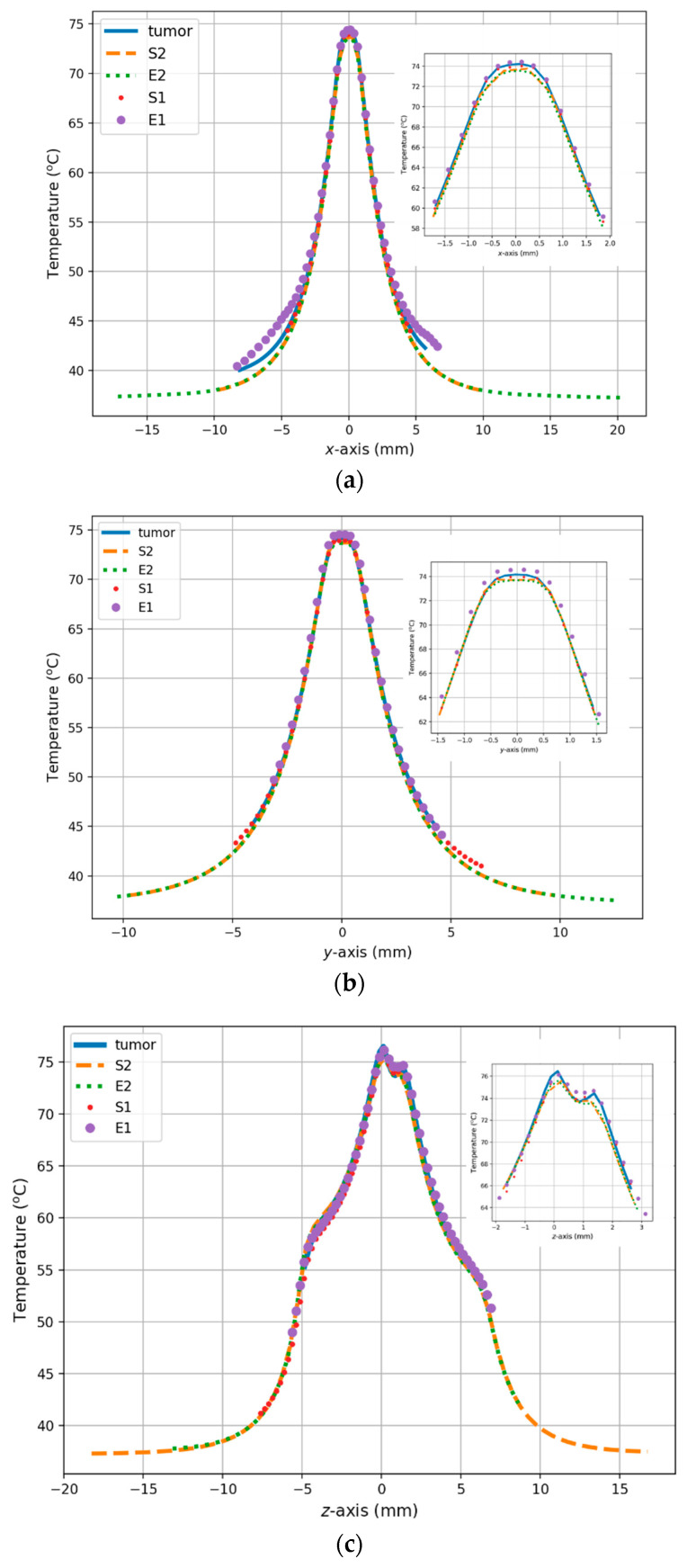
Temperature distributions along different axes of analyzed equivalent tumor models: (**a**) *x*-axis; (**b**) *y*-axis; (**c**) *z*-axis, where S_1_ and S_2_ are spherically equivalent tumor models; and E_1_ and E_2_ ellipsoidal equivalent tumor models.

**Figure 9:**
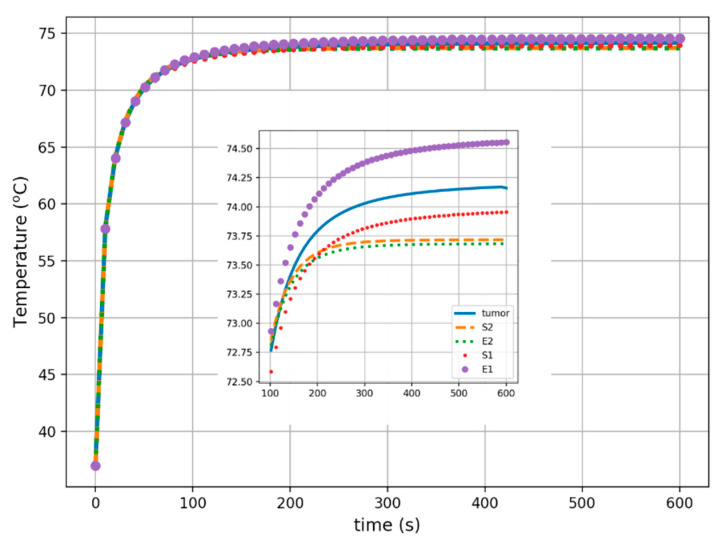
Time-dependent temperature distributions in the center of various tumors, including zoom view inside, where S_1_ and S_2_ correspond to spherical tumor models and E_1_ and E_2_ correspond to ellipsoidal tumor models.

The last two figures illustrate the spatial and temporal temperature profiles for all cases considered. Figure 8 shows the tumor temperature characteristics along the main axes of the Cartesian system (*x*, *y*, *z*). All curves refer to the mass center of tumor models, which was set as the coordinate origin point. Inside the tumor models, the heat profiles converge well along a distance of about 4 mm from the center of the tumors (for the *x*- and *y*-axes) and even 18 mm along the *z*-axis. Finally, the temperature over time in the center of the analyzed tumor models is presented in Figure 9. The obtained tumor thermal profiles indicated that a steady state was reached after about 300 s.

**Figure 10:**
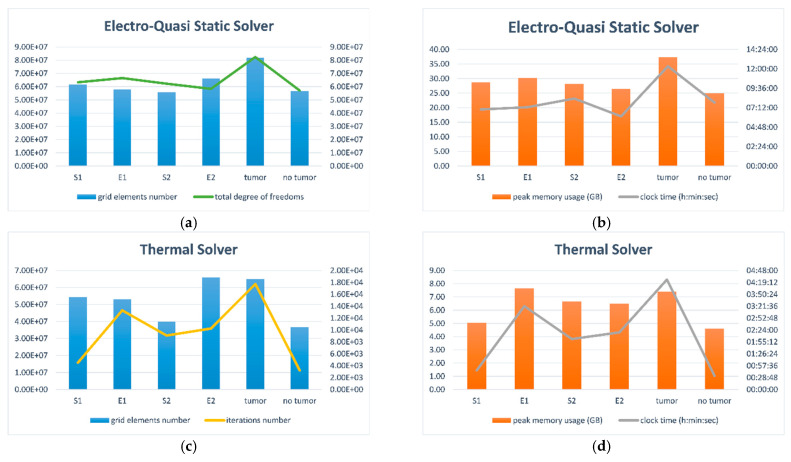
Grid element number, total degree of freedoms, peak memory usage, and clock time values for an electro-quasi-static solver (**a**,**c**); and a thermal solver (**b**,**d**) in the case of all analyzed tumor models.

The anatomically correct model of the female breast with a naturalistic tumor was created at a huge computational cost. Figure 10 compares all analyzed tumor models separately for each solver. In the case of the Electro-Quasi-Static Solver, the grid element number, the total degree of freedom, the peak memory usage, and the computing time were taken into account. The following parameters were compiled in the case of the Thermal Solver: the grid element number, the interaction number, the peak memory usage, and the computing time. The vertical bars in Figure 10 correspond to the left *y*-axis, while the solid line corresponds to the right *y*-axis.

The figures presented above show that the breast phantom with naturalistic tumor T was the most computationally demanding model (total computing time: 16 h 45 min 25 s; total degree of freedom 82,463,040), whereas the phantom without tumor was the least computationally demanding (total computing time: 8 h 22 min 53 s; total degree of freedom 57,132,348). The other models, which included the equivalent tumor models, exhibited the following intermediate values: ellipsoid E1 (total degree of freedom 58,399,380), most similar in terms of geometric parameters to the naturalistic tumor T, was solved within a total computing time of 10 h 38 min 8 s, which gave a faster solution by more than 6 h than the tumor T model. The breast model with ellipsoid E2 (total degree of freedom 66,533,376) was solved within a total computing time of 8 h 26 min 52 s, and the models with equivalent spheres S1 (total degree of freedom 62,252,940) and S2 (total degree of freedom 63,399,040) were solved within 7 h 45 min 51 s and 10 h 22 min 18 s, respectively. The following homogeneous meshes were used: breast model—voxel size 1 mm × 1 mm × 1 mm; tumor models—voxel size 0.5 mm × 0.5 mm × 0.5 mm; electrode 1, electrode 2, and dielectric 2–0.3 mm × 0.3 mm × 0.3 mm; dielectric 1 and catheter—voxel size 0.5 mm × 0.5 mm × 0.5 mm (see Figure 4). The generated meshes can be considered dense. Detailed information regarding quasi-static approximation was included in the Supplementary Materials. The shortened computational time may be a great benefit of using equivalent tumor models.

## 4. Discussion

In earlier studies of numerical hyperthermia and thermal ablation procedures, spherical [29,49] or ellipsoidal tumor models [43,44] were considered most frequently. Additionally, irregular tumor models were analyzed [9,54]. The spherical and ellipsoidal tumor shapes constitute the vast majority of possible shapes of female breast tumors used in computational practice. However, it is still being debated whether the irregularly shaped tumor can be replaced with a regular one.

For example, 2D mathematical modeling of heating a spherically shaped breast tumor during RF ablation was considered in [49]. The needle-type RF applicator with a voltage ranging from 10 to 20 V was used, and the temperature in the spherical tumor was analyzed. The naturalistic 3D model of the female breast with spherical tumor was modeled in [29]. However, as can be found in [5,6], the vast majority of breast cancers do not have a spherical shape.

In another study [43], a theoretical analysis of magnetic hyperthermia of ellipsoidal tumors with various shapes (different aspect ratio, AR, values for oblate and prolate tumors) immersed in breast tissue was reported. The analysis was conducted for frequency *f* = 220 kHz and amplitude of magnetic field strength *H* = 6.8 kA/m. It was concluded that increasing AR results in a temperature decrease in the center of both prolate and oblate ellipsoidal tumor shapes.

There is no link with any specific case of tumor in the papers mentioned above, and the reported cases were randomly chosen. There is also no methodology that tries to effectively reflect tumor shapes.

Breast tumors with irregular shapes were analyzed in [9], where 22 MRI-based models of breasts with cancers were created. This relatively large repository can be used for the planning, evaluation, and development of treatment, but there are still special cases of breast tumors. In terms of size, location, and tumor depth, the repository covers the majority of breast tumors in the T1, T2, and T3 stages statistically. However, generalized tumor models may be required to ensure the efficiency of treatment strategies for a wider patient population.

The equivalent female breast tumor models proposed in this study facilitate a reliable and representative evaluation of various types of tumors in RF ablation treatment.

As indicated by the computed SAR distributions for all models presented in Figure 6 and quantified in Table 3, the maximum, mass-average, and spatial-average SAR values are comparable in the T, E1, and S1 models. This result indicates SAR changes lower than 10%. However, the presented SAR analysis does not indicate which equivalent tumor model was ablated most effectively compared with tumor T. The data collected in Table 3 show that the power deposition levels for tumor T are similar to those for the S1-equivalent tumor model. A slightly worse agreement was found in the case of model E1. The worst results were recorded for the E2- and S2-equivalent models, which were associated with their much larger volumes than the naturalistic tumor T size.

As demonstrated by the SAR analysis, the S1- and E1-equivalent tumor models, with a similar volume and weight to that of the naturalistic tumor T, yielded SAR values that were the closest to those of the referenced model of the irregular tumor T. The best convergence in the SAR analysis was obtained in the case of the S1-equivalent model. The simulated SAR distributions for equivalent tumor models, defined as sphere S2 and ellipsoid E2, which surrounded the irregular tumor, were slightly worse. This was mainly related to their much larger volumes than the original tumor T, resulting in much lower levels of volumetric power density dissipated in these models.

The isosurface temperature patterns (see Figure 7) demonstrate that the probe with a specific voltage placed in the tumor-free female breast phantom produces almost spherical isosurfaces. In the presence of the tumor, the isosurfaces become ablated in the RF applicator axis direction (*z*-axis). From the therapeutic point of view, temperatures above 50 °C (isosurface-50) can be regarded as ablated zones [54]. Table 4 lists the ablative volumes (limited by isosurface-50) of all the breast tumor models analyzed. The ablation zones were found to have similar values. The largest and the smallest ablation zones were found in the E1-equivalent tumor model (*V*_ISO-50_ = 242.536 mm^3^) and in the ellipsoid E2 tumor model (*V*_ISO-50_ = 201.253 mm^3^), respectively. However, the closest ablation value to the tumor model T (*V*_ISO-50_ = 223.215 mm^3^) was achieved by the S1-equivalent tumor model (*V*_ISO-50_ = 226.064 mm^3^). Considering the volume of the ablation zone of tumor T as a reference, it can be seen that the ablative zones did not change considerably (less than a 10% variation in volume). This result may suggest that the effectiveness of ablation does not depend on the utilized tumor model but mainly on the RF probe applied; therefore, equivalent tumor models can be used instead of irregularly shaped tumors.

The temporal and spatial analyses were performed to complement the temperature analysis. The temporal thermal analysis showed that the highest temperature inside the tumor, and in the tumor center, was induced in the E1-equivalent tumor model (74.6 °C), and was 0.4 °C higher than the temperature of the naturalistic tumor T. The temperature inside the S1-equivalent tumor model was lower than that of the irregular tumor T, but the difference in the temperature in this case was lower by approximately 0.3 °C. This might suggest that the volume of the equivalent models does not considerably influence the temperature rise.

The spatial distribution shown in Figure 8 indicates the most efficient heating in the E1-equivalent model and tumor T and the lowest efficiency of the process in the E2- and S2-equivalent tumors. The differences in the temperatures inside the tumors result from the different shapes and volumes of the particular model. The temperature differences at the periphery of the tumors are a direct result of the assumed sizes and shapes of the tumors, as well as the distribution of different tissues around the tumor and their different perfusion rates. This effect can be seen in Figure 9, which shows the changes in temperature over time in the models under consideration. For example, the steady-state temperature after 300 s was approximately 74.6 °C in the center of the E1-equivalent tumor model and 74.2 °C inside the irregular tumor T. In the case of the S2- and E2-equivalent models, the steady-state temperature reached the same temperature of 73.7 °C.

It can be concluded that both S1- and E1-equivalent tumor models can be successfully used to model real irregular tumors. All models successfully reduced the computing power and time, reaching over 2 h acceleration in the E1 model. Therefore, this model may be considered the most suitable for estimating tumor temperature distribution patterns in the case of RF ablation treatment planned with the use of the proposed methodology. It was shown that there was a negligible difference in the predicted SAR patterns and ablated zones between the naturalistic tumor model and its equivalent.

The limitations of the proposed methodology result from the assumed mathematical model, i.e., the tissue parameters were taken from the actual databases based on the performed experimental measurements; the uniform, linear, and isotropic breast tissue parameters were assumed, with the exception of the tumor models, where a nonlinear blood perfusion model was considered. Thus, the proposed approach needs further modifications based on both experimental and numerical results.

## 5. Conclusions

In this study, four equivalent models simplifying the breast tumor (two spherical and two elliptical) were tested to find out the effect of the complexity of the tumor model on the levels of power dissipation and heating in cancerous tissue. A novelty of the present work was the creation of a numerical platform including an anatomical model of a female breast with a naturalistic, irregularly shaped tumor. The analysis of power density, SAR, and temperature distribution revealed that the irregularly shaped tumor could be replaced by an equivalent model with a similar volume and mass, resulting in almost the same ablation zone and a shorter calculation time. It seems that an ellipsoid is the most efficient alternative for a naturalistic tumor.

Although the numerical phantom was created for a specific medical case of a female breast with an irregular tumor, the presented methodology and proposed numerical platform can be applied to analyze female breast phantoms with differently shaped tumors effectively. It is obvious that it is necessary to modify the adopted model with the results of actual measurements of the tissues of a particular patient. For this purpose, several in vivo clinical experiments should be conducted in the future, and their results should be compared with the results obtained from the numerical platform. Then, this platform should be modified appropriately to be successfully used in clinical applications.

## Figures and Tables

**Table 1:** Electro-thermal female breast tissue parameters valid for RF ablation treatment with frequency *f* = 100 kHz [60].

Tissue	*σ* (S/m)	*ρ* (kg/m^3^)	*C* (J/kg/K)	*k* (W/m/K)	HTR * (mL/min/kg)	HTR * (W/m^3^)	HGR ** (W/kg)
Blood	0.7030	1050	3617	0.517	10,000	6.646 × 10^5^	0
Breast fat	0.0250	911	2348	0.209	47	2710	0.728
Breast gland	0.5370	1041	2960	0.334	150	9884	2.323
Fat	0.0434	911	2348	0.211	33	1903	0.507
Muscle	0.3618	1090	3421	0.495	37	2553	0.906
Skin	0.0005	1109	3391	0.372	106	7441	1.648
Tumor	0.3618	1090	3437	0.563	Equation (9)	Equation (9)	12

* Heat Transfer Rate, ** Heat Generation Rate.

**Table 2:** Geometric parameters for different shaped tumors studied.

Breast Tumor Tissues	*x-*axis Length *a* (mm)	*y-*axis Length *b* (mm)	*z-*axis Length *c* (mm)	Total Surface Area *A*_calc_ (cm^2^)	Total Surface Area *A*_meas_ (cm^2^)	Total Volume *V*_calc_ (cm^3^)	Total Volume *V*_meas_ (cm^3^)	Total Mass *m*_calc_ (g)	Total Mass *m*_meas_ (g)
Ellipsoid E_1_	16.412	29.354	10.866	13.153	12.716 *	2.740	2.749 *	2.989	2.998 *
Sphere S_1_	17.474	17.474	17.474	9.593	9.538 *	2.794	2.771 *	3.046	3.021 *
Ellipsoid E_2_	26.574	42.588	27.675	31.498	32.153 *	16.395	16.310 *	17.882	17.790 *
Sphere S_2_	36.000	36.000	36.000	40.715	40.484 *	24.429	24.270 *	26.637	26.460 *
Tumor T	38.630	24.269	24.269	12.320 **	12.722 *	2.844 **	2.791 *	3.101 **	3.043 *

* data measured from the mesh; ** data calculated for equivalent cube.

**Table 3:** Calculated power losses inside breast tissues during RF ablation treatment with frequency *f* = 100 kHz.

Breast Tissues	Total Mass *m* (g)	Total Volume *V* (cm^3^)	Maximum Local SAR SAR_max_ (W/kg)	Mass-Average Local SAR SAR_mass_ (W/kg)	Total Loss Power Density *p* = SAR_vol_ (W/m^3^)	Spatial-Average Local SAR ** psSAR_1g_ (W/kg)	Total Loss Power *P* (W)
Breast fat *	4766.2	5232.1	9560	4.95 × 10^−3^	13.525	650.82	0.0254
Breast gland *	814.8	783.0	2,7440	7.099	7388.65	699.33	1.1485
Fat *	441.3	484.4	11,430	4.41 × 10^−2^	40.153	1050.0	0.0195
Muscle *	731.3	670.6	1.61 × 10^−6^	8.24 × 10^−7^	8.99 × 10^−4^	1.58 × 10^−6^	6.0 × 10^−7^
Skin *	495.6	446.9	4.10 × 10^−4^	8.31 × 10^−7^	9.22 × 10^−4^	0.142	19.524
Ellipsoid E_1_	2.998	2.749	152,700	326.435	355 984	823.41	0.9786
Sphere S_1_	3.021	2.771	151,900	327.576	357,163	825.05	0.9897
Ellipsoid E_2_	17.79	16.31	155,600	58.844	64,175	833.98	1.0467
Sphere S_2_	26.46	24.27	155,800	39.733	43,325	835.40	1.0515
Tumor T	3.043	2.791	167,200	327.894	357,506	824.83	0.9978

* data for model without tumor, ** SAR value averaged by 1 g mass of tissue defined by IEEE/IEC62704-1 standard [65].

**Table 4:** Calculated ablation volumes and relative errors in the equivalent tumor models.

Breast Tissues	Ablation Volume *V*_ISO-50_ (mm^3^)	Relative Error *δ* (%)
Ellipsoid E_1_	242.536	8.656
Sphere S_1_	226.064	1.276
Ellipsoid E_2_	201.253	9.839
Sphere S_2_	204.365	8.445
Tumor T	223.215	–

## Data Availability

Not applicable.

## Supplementary Materials

The following supporting information can be downloaded at: https://www.mdpi.com/article/10.3390/ma16010223/s1, Table S1: The quasi-static approximation criteria for the Electro-Quasi-Static Solver (EQS) for *f* = 100 kHz.

## References

[1] Barba D., Leon-Sosa A., Lugo P., Suquillo D., Torres F., Surre F., Trojman L., Caicedo A. (2021). Breast cancer, screening and diagnostic tools: All you need to know. Crit. Rev. Oncol. Hematol..

[2] Pelicano A.C., Gonçalves M.C.T., Godinho D.M., Castela T., Orvalho M.L., Araújo N.A.M., Porter E., Conceicao R.C. (2021). Development of 3D MRI-Based Anatomically Realistic Models of Breast Tissues and Tumours for Microwave Imaging Diagnosis. Sensors.

[3] Korczak I., Romowicz A., Gambin B., Palko T., Kruglenko E., Dobruch-Sobczak K. (2020). Numerical prediction of breast skin temperature based on thermographic and ultrasonographic data in healthy and cancerous breasts. Biocybern. Biomed. Eng..

[4] Osmialowska E., Misiag W., Chabowski M., Jankowska-Polanska B. (2021). Coping strategies, pain, and quality of life in patients with breast cancer. J. Clin. Med..

[5] Byrd B.K., Krishnaswamy V., Gui J., Rooney T., Zuurbier R., Rosenkranz K., Paulsen K., Barth R.J. (2020). The shape of breast cancer. Breast Cancer Res. Treat..

[6] Krekel N., Zonderhuis B., Muller S., Bril H., van Slooten H.-J., de Lange de Klerk E., van den Tol P., Meijer S. (2011). Excessive Resections in Breast-Conserving Surgery: A Retrospective Multicentre Study. Breast J..

[7] Uematsu T., Kasami M., Yuen S. (2009). Triple-negative breast cancer: Correlation between MR imaging and pathologic findings. Radiology.

[8] Wapnir I.L., Wartenberg D.E., Greco R.S. (1996). Three dimensional staging of breast cancer. Breast Cancer Res. Treat..

[9] Androulakis I., Sumser K., Machielse M.N., Koppert L., Jager A., Nout R., Franckena M., van Rhoon G.C., Curto S. (2022). Patient-derived breast model repository, a tool for hyperthermia treatment planning and applicator design. Int. J. Hyperth..

[10] Burguin A., Diorio C., Durocher F. (2021). Breast cancer treatments: Updates and new challenges. J. Pers. Med..

[11] Moath A., Xiao Y.X. (2021). The influence of tumour vasculature on fluid flow in solid tumours: A mathematical modelling study. Biophys. Rep..

[12] Eltigani F., Ahmed S., Yahya M., Ahmed M. (2022). Modeling of interstitial microwave hyperthermia for hepatic tumors using floating sleeve antenna. Phys. Eng. Sci. Med..

[13] Sano M.B., DeWitt M.R., Teeter S.D., Xing L. (2018). Optimization of a single insertion electrode array for the creation of clinically relevant ablations using high-frequency irreversible electroporation. Comput. Biol. Med..

[14] de los Ríos Cardenas L., Bermeo L.A., de Albuquerque Pereira W.C. (2020). Parameter estimation in high-intensity focused ultrasound therapy. Int. J. Numer. Methods Biomed. Eng..

[15] Tammam E., Said A.M., Ibrahim A.A., Galal A.I. (2020). About the interstitial microwave cancer ablation: Principles, advantages and challenges. IEEE Access.

[16] Luyen H., Mohtashami Y., Sawicki J.F., Hagness S.C., Behdad N., Rahmat-Samii Y., Topsakal E. (2021). Minimally Invasive Microwave Ablation Antennas. Antenna and Sensor Technologies in Modern Medical Applications.

[17] Ramirez-Guzman T.J., Trujillo-Romero C.J., Martinez-Valdez R., Leija-Salas L., Vera-Hernandez A., Rico-Martinez G., Ortega-Palacios R., Gutierrez-Martinez J. (2021). Thermal Evaluation of a Micro-Coaxial Antenna Set to Treat Bone Tumors: Design, Parametric FEM Modeling and Evaluation in Multilayer Phantom and Ex Vivo Porcine Tissue. Electronics.

[18] Ashour A.S., Guo Y., Mohamed W.S. (2021). Ablation probes. Thermal Ablation Therapy, Theory and Simulation.

[19] Preechaphonkul W., Rattanadecho P. (2021). The comparative of the performance for predicted thermal models during microwave ablation process using a slot antenna. Case Stud. Therm. Eng..

[20] Huang H., Zhang L., Moser M.A., Zhang W., Zhang B. (2021). A review of antenna designs for percutaneous microwave ablation. Phys. Med..

[21] Gas P., Szymanik B. Shape optimization of the multi-slot coaxial antenna for local hepatic heating during microwave ablation. Proceedings of the 2018 International Interdisciplinary PhD Workshop (IIPhDW).

[22] Geoghegan R., Ter Haar G., Nightingale K., Marks L., Natarajan S. (2022). Methods of monitoring thermal ablation of soft tissue tumors—A comprehensive review. Med. Phys..

[23] Barnoon P., Ashkiyan M. (2020). Magnetic field generation due to the microwaves by an antenna connected to a power supply to destroy damaged tissue in the liver considering heat control. J. Magn. Magn. Mater..

[24] Nizam-Uddin N., Abdulkawi W.M., Elshafiey I., Sheta A.F.A. (2022). Towards an efficient system for hyperthermia treatment of breast tumors. Biomed. Signal Process. Control.

[25] Gas P., Miaskowski A., Subramanian M. (2020). In silico study on tumor-size-dependent thermal profiles inside an anthropomorphic female breast phantom subjected to multi-dipole antenna array. Int. J. Mol. Sci..

[26] Hassan M.M., Lias K., Buniyamin N., Naimullah B.S.S., Jobli A.T. (2021). SAR Performance of Rectangular Microstrip Antenna for Breast Cancer Hyperthermia Treatment with Different Period of Treatment Procedure. J. Phys. Conf. Ser..

[27] Gas P., Wyszkowska J. (2019). Influence of multi-tine electrode configuration in realistic hepatic RF ablative heating. Arch. Elect. Eng..

[28] Barnoon P., Bakhshandehfard F. (2021). Thermal management in biological tissue in order to degrade tissue under local heating process. Case Stud. Therm. Eng..

[29] Rahpeima R., Lin C.-A. (2022). Numerical Study of Magnetic Hyperthermia Ablation of Breast Tumor on an Anatomically Realistic Breast Phantom. PLoS ONE.

[30] Li J., Xu Y., Shen N., Feng L., Ran Z., Deng Z. (2020). A practical pretreatment planning method of multiple puncturing for thermal ablation surgery. Biocybern. Biomed. Eng..

[31] Liu P., Qin J., Duan B., Wang Q., Tan X., Zhao B., Peneyra Libao J., Chui C.-K., Heng P.A. (2019). Overlapping radiofrequency ablation planning and robot-assisted needle insertion for large liver tumors. Int. J. Med. Robot. Comput. Assist. Surg..

[32] Nee Reimann C.H., Bazrafshan B., Schüßler M., Schmidt S., Schuster C., Hübner F., Vogl T.J., Jakoby R. (2018). A dual-mode coaxial slot applicator for microwave ablation treatment. IEEE Trans. Microw. Theory Tech..

[33] Ashour A.S., Asran M., Mohamed W.S., Fotiadis D.I. (2021). Optimal Localization of a Novel Shifted 1T-Ring Based Microwave Ablation Probe in Hepatocellular Carcinoma. IEEE. Trans. Biomed. Eng..

[34] Palandoken M., Murat C., Kaya A., Zhang B. (2022). A Novel 3-D Printed Microwave Probe for ISM Band Ablation Systems of Breast Cancer Treatment Applications. IEEE Trans. Microw. Theory Tech..

[35] Portosi V., Loconsole A.M., Valori M., Marrocco V., Fassi I., Bonelli F., Pascazio G., Lampignano V., Fasano A., Prudenzano F. (2021). Low-Cost Mini-Invasive Microwave Needle Applicator for Cancer Thermal Ablation: Feasibility Investigation. IEEE Sens. J..

[36] Wang J., Huang S., Gao H., Liu J., Zhang Y., Wu S. (2023). Computer Simulations of Dual-Antenna Microwave Ablation and Comparison to Experimental Measurements. Appl. Sci..

[37] Ferrero R., Barrera G., Celegato F., Vicentini M., Sozeri H., Yildiz N., Dincer C.A., Coisson M., Manzin A., Tiberto P. (2021). Experimental and Modelling Analysis of the Hyperthermia Properties of Iron Oxide Nanocubes. Nanomaterials.

[38] Raouf I., Gas P., Kim H.S. (2021). Numerical Investigation of Ferrofluid Preparation during In-Vitro Culture of Cancer Therapy for Magnetic Nanoparticle Hyperthermia. Sensors.

[39] Szczech M. (2020). Magnetic fluid seal critical pressure calculation based on numerical simulations. Simulation.

[40] Yu X., Ding S., Yang R., Wu C., Zhang W. (2021). Research progress on magnetic nanoparticles for magnetic induction hyperthermia of malignant tumor. Ceram. Int..

[41] Suleman M., Riaz S., Jalil R. (2021). A mathematical modeling approach toward magnetic fluid hyperthermia of cancer and unfolding heating mechanism. J. Therm. Anal. Calorim..

[42] Tang Y., Su H., Flesch R.C., Jin T. (2022). An optimization method for magnetic hyperthermia considering Nelder-Mead algorithm. J. Magn. Magn. Mater..

[43] Polychronopoulos N.D., Gkountas A.A., Sarris I.E., Spyrou L.A. (2021). A Computational Study on Magnetic Nanoparticles Hyperthermia of Ellipsoidal Tumors. Appl. Sci..

[44] Tehrani M.H., Soltani M., Kashkooli F.M., Raahemifar K. (2020). Use of microwave ablation for thermal treatment of solid tumors with different shapes and sizes—A computational approach. PLoS ONE.

[45] Segura Felix K., Lopez G., Geshel D., Cepeda Rubio M.F., Hernandez Jacquez J.I., Flores Garcia F.G., Vera Hernandez A., Leija Salas L., Orozco Ruiz de la Pena E.C. (2021). Computational FEM Model and Phantom Validation of Microwave Ablation for Segmental Microcalcifications in Breasts Using a Coaxial Double-Slot Antenna. BioMed Res. Int..

[46] Lim S., Yoon Y.J. (2021). Phase Compensation Technique for Effective Heat Focusing in Microwave Hyperthermia Systems. Appl. Sci..

[47] Kalogeropoulos A., Tsitsas N.L. (2021). Analysis of Interaction Scattering Cross Sections and Their Physical Bounds for Multiple-Dipole Stimulation of a Three-Dimensional Layered Medium. IEEE Trans. Antennas Propag..

[48] Prokhorova A., Ley S., Helbig M. (2021). Quantitative Interpretation of UWB Radar Images for Non-Invasive Tissue Temperature Estimation during Hyperthermia. Diagnostics.

[49] Paruch M. (2020). Mathematical Modeling of Breast Tumor Destruction using Fast Heating during Radiofrequency Ablation. Materials.

[50] Rajput J.L., Nandgaonkar A.B., Nalbalwar S.L., Wagh A.E. (2021). Heat Flow Modeling for Controlled Focusing of Microwave Hyperthermia of Breast Cancer: A Computational Feasibility Study. Int. J. Adv. Sci. Eng. Inf. Technol..

[51] Michalowska-Samonek J., Miaskowski A., Wac-Wlodarczyk A. (2012). Numerical analysis of high frequency electromagnetic field distribution and specific absorption rate in realistic breast models. Prz. Elektrotechniczny.

[52] Sawicki B., Miaskowski A. (2014). Nonlinear higher-order transient solver for magnetic fluid hyperthermia. J. Comput. Appl. Math..

[53] Cao T.L., Le T.A., Hadadian Y., Yoon J. (2021). Theoretical Analysis for Using Pulsed Heating Power in Magnetic Hyperthermia Therapy of Breast Cancer. Int. J. Mol. Sci..

[54] Radjenovic B., Sabo M., Soltes L., Prnova M., Cicak P., Radmilovic-Radjenovic M. (2021). On Efficacy of Microwave Ablation in the Thermal Treatment of an Early-Stage Hepatocellular Carcinoma. Cancers.

[55] Phantom Repository University of Wisconsin Cross-Disciplinary Electromagnetics Laboratory. https://uwcem.ece.wisc.edu/phantomRepository.html.

[56] Zastrow E., Davis S.K., Lazebnik M., Kelcz F., Van Veen B.D., Hagness S.C. (2008). Development of anatomically realistic numerical breast phantoms with accurate dielectric properties for modeling microwave interactions with the human breast. IEEE Trans. Biomed. Eng..

[57] Gas P., Miaskowski A., Dobrowolski D. (2020). Modelling the tumor temperature distribution in anatomically correct female breast phantom. Prz. Elektrotechniczny.

[58] Koh J., Kim M.J. (2019). Introduction of a New Staging System of Breast Cancer for Radiologists: An 614 Emphasis on the Prognostic Stage. Korean J. Radiol..

[59] Gabriel S., Lau R.W., Gabriel C. (1996). The dielectric properties of biological tissues: III. Parametric models for the dielectric spectrum of tissues. Phys. Med. Biol..

[60] Hasgall P.A., Di Gennaro F., Baumgartner C., Neufeld E., Lloyd B., Gosselin M.C., Payne D., Klingenbock A., Kuster N. IT’IS Database for Thermal and Electromagnetic Parameters of Biological Tissues, Version 4.1, 22 February 2022. https://itis.swiss/database.

[61] Pennes H.H. (1998). Analysis of Tissue and Arterial Blood Temperatures in the Resting Human Forearm. J. Appl. Physiol..

[62] Szasz O., Szasz A. (2021). Approaching Complexity: Hyperthermia Dose and its Possible Measurement in Oncology. Open J. Biophys..

[63] Michalowska J., Wac-Wlodarczyk A., Koziel J. (2020). Monitoring of the Specific Absorption Rate in Terms of Electromagnetic Hazards. J. Ecol. Eng..

[64] Wainwright P.R. (2003). The relationship of temperature rise to specific absorption rate and current in the human leg for exposure to electromagnetic radiation in the high frequency band. Phys. Med. Biol..

[65] IEC/IEEE 62704-1:2017, Determining the Peak Spatial-Average Specific Absorption Rate (SAR) in the Human Body from Wireless Communications Devices, 30 MHz to 6 GHz—Part 1: General Requirements for Using the Finite Difference Time-Domain (FDTD) Method for SAR Calculations. https://standards.ieee.org/ieee/62704-1/5747/.

[66] Kurazumi Y., Tsuchikawa T., Ishii J., Fukagawa K., Yamato Y., Matsubara N. (2008). Radiative and convective heat transfer coefficients of the human body in natural convection. Build Environ..

[67] Prasad B., Ha Y.H., Lee S.K., Kim J.K. (2016). Patient-specific simulation for selective liver tumor treatment with noninvasive radiofrequency hyperthermia. J. Mech. Sci. Technol..

