# Supplementary material for: Numerical Estimation of SAR and Temperature Distributions inside Differently Shaped Female Breast Tumors during Radio-Frequency Ablation
Source: Materials (Basel). 2022 Dec 26;16(1):223. doi: 10.3390/ma16010223 (PMC9821952; doi:10.3390/ma16010223)
Supplement: Supplementary file 1 [file materials-16-00223-s001.zip › materials-2076080-supplementary.pdf]

materials MDPI

Supplementary Materials

Numerical Estimation of SAR and Temperature Distributions inside Differently Shaped Female Breast Tumors during Radio-Frequency Ablation

Arkadiusz Miaskowski [1,*] and Piotr Gas [2,*]

[1] Department of Applied Mathematics and Computer Sciences, Faculty of Production Engineering, University of Life Sciences in Lublin, Akademicka 13 Street, 20-950 Lublin, Poland
[2] Department of Electrical and Power Engineering, Faculty of Electrical Engineering, Automatics, Computer Science and Biomedical Engineering, AGH University of Science and Technology, Mickiewicza 30 Avenue, 30-059 Krakow, Poland
* Correspondence: arek.miaskowski@up.lublin.pl (A.M.); piotr.gas@agh.edu.pl (P.G.)

1. Numerical Modelling

Table S1. The quasi-static approximation criteria for the Electro-Quasi Static Solver (EQS) for $f = 100$ kHz.

Material	Epsilon	Mu	SigmaE	QS Approx. Quality	Static Magnetic Field	SigmaE/w*Epsilon
Background (Air)	1	1	0	1.7462×10^{-6}	0	0
Fatty-1	70.6	1	0.025	0.0001233	0.0078625	63.766
Fatty-2	70.6	1	0.025	0.0001233	0.0078625	63.766
Fatty-3	70.6	1	0.025	0.0001233	0.0078625	63.766
Fibroconnective/glandular-1	3.3×10^3	1	0.537	0.0057646	0.16855	29.239
Fibroconnective/glandular-2	3.3×10^3	1	0.537	0.0057646	0.16855	29.239
Fibroconnective/glandular-3	3.3×10^3	1	0.537	0.0057646	0.16855	29.239
Muscle	8.09×10^3	1	0.362	0.014126	0.11358	8.0407
Skin	1.12×10^3	1	0.000451	0.0019544	0.00014165	0.072479
Transitional	101	1	0.0434	0.00017723	0.013623	76.867
Tumor	8.09×10^3	1	0.362	0.014126	0.11358	8.0407
Dielectric 1 (Applicator)	2.25	1	0.0005	3.929e-06	0.00015694	39.945
Dielectric 2 (Applicator)	2.25	1	0.0005	3.929e-06	0.00015694	39.945
Catheter (Applicator)	2.25	1	0.0005	3.929e-06	0.00015694	39.945

*The column QS Approx. Quality and Static Magnetic Field refers to quasi-static conditions. The column SigmaE/w*Epsilon indicates whether the displacement current ($\ll 1$) or the ohmic current dominates ($\gg 1$).*
